# Fibroblast Growth Factor Receptor 1 Signaling in Adult Cardiomyocytes Increases Contractility and Results in a Hypertrophic Cardiomyopathy

**DOI:** 10.1371/journal.pone.0082979

**Published:** 2013-12-11

**Authors:** Sarah N. Cilvik, Joy I. Wang, Kory J. Lavine, Keita Uchida, Angela Castro, Carolyn M. Gierasch, Carla J. Weinheimer, Stacey L. House, Attila Kovacs, Colin G. Nichols, David M. Ornitz

**Affiliations:** 1 Department of Developmental Biology, Washington University School of Medicine, St. Louis, Missouri, United States of America; 2 Department of Internal Medicine, Washington University School of Medicine, St. Louis, Missouri, United States of America; 3 Department of Cell Biology and Physiology, Washington University School of Medicine, St. Louis, Missouri, United States of America; 4 Division of Emergency Medicine, Washington University School of Medicine, St. Louis, Missouri, United States of America; Scuola Superiore Sant'Anna, Italy

## Abstract

Fibroblast growth factors (FGFs) and their receptors are highly conserved signaling molecules that have been implicated in postnatal cardiac remodeling. However, it is not known whether cardiomyocyte-expressed FGF receptors are necessary or sufficient for ventricular remodeling in the adult heart. To determine whether cardiomyocytes were competent to respond to an activated FGF receptor, and to determine if this signal would result in the development of hypertrophy, we engineered a doxycycline (DOX)-inducible, cardiomyocyte-specific, constitutively active FGF receptor mouse model (*αMHC-rtTA, TRE-caFgfr1-myc*). Echocardiographic and hemodynamic analysis indicated that acute expression of caFGFR1 rapidly and directly increased cardiac contractility, while chronic expression resulted in significant hypertrophy with preservation of systolic function. Subsequent histologic analysis showed increased cardiomyocyte cross-sectional area and regions of myocyte disarray and fibrosis, classic features of hypertrophic cardiomyopathy (HCM). Analysis of downstream pathways revealed a lack of clear activation of classical FGF-mediated signaling pathways, but did demonstrate a reduction in Serca2 expression and troponin I phosphorylation. Isolated ventricular myocytes showed enhanced contractility and reduced relaxation, an effect that was partially reversed by inhibition of actin-myosin interactions. We conclude that adult cardiomyocytes are competent to transduce FGF signaling and that FGF signaling is sufficient to promote increased cardiomyocyte contractility *in vitro* and *in vivo* through enhanced intrinsic actin-myosin interactions. Long-term, FGFR overexpression results in HCM with a dynamic outflow tract obstruction, and may serve as a unique model of HCM.

## Introduction

Hypertrophy is an adaptive mechanism by which the heart can respond to stress or injury. In response to postnatal growth, pregnancy, or exercise, the heart undergoes physiological hypertrophy, characterized by an increase in myocyte length, absence of fibrosis, maintenance of chamber dimension, and normal cardiac function. In response to injury or stress, such as a myocardial infarction or increased afterload, functional heart muscle compensates with pathological hypertrophy, which initially maintains cardiac function. Pathological hypertrophy is characterized by increased myocyte width, fibrosis, decreased ventricular chamber volume, and eventual cardiac dysfunction and failure [1,2]. Additionally, hypertrophic cardiomyopathy (HCM), a primary myocardial disorder, is most often caused by dominant mutations in sarcomeric proteins and characterized by myocyte hypertrophy and disarray, fibrosis, and maintenance of systolic function [3,4]. Many studies have attempted to elucidate mechanisms that result in hypertrophy, but this process remains poorly understood, particularly with regard to mechanisms that distinguish advantageous physiological hypertrophy from detrimental pathological hypertrophy.

Numerous studies have implicated the Fibroblast Growth Factor (FGF) family in the development of postnatal cardiac hypertrophy. Addition of FGF2 to cultured neonatal rat cardiomyocytes repressed adult cardiac genes and induced embryonic genes, an expression profile characteristic of pressure overload-induced hypertrophy [5–9]. Furthermore, the hypertrophy exhibited by paced adult rat cardiomyocytes was prevented by an anti-FGF2 antibody [10]. Thus, it is not surprising that mice lacking FGF2 develop significantly less hypertrophy than wild-type mice after being subjected to pressure overload by transverse aortic constriction (TAC) [11]. Many other studies have demonstrated that FGF2 is both necessary and sufficient for the development of pathological hypertrophy following injury. Systemic administration of FGF2 following acute myocardial infarction in rats induced significant hypertrophy in non-infarcted myocardium [12]. Similarly, another study demonstrated that Fgf2^-/-^ hearts have decreased cardiomyocyte hypertrophy and impaired cardiac function compared to WT mice following MI, while FGF2-overexpressing hearts showed enhanced cardiomyocyte hypertrophy and preserved function [13]. Additionally, knockout of *Fgf2* prevented the development isoproterenol- and angiotensin II-induced pathological hypertrophy in the adult heart, while transgenic overexpression of FGF2 in the heart showed an exacerbated hypertrophic response to β-adrenergic stimulation, an effect mediated by signaling through ERK1/2 [14,15].

In addition to FGF2, FGF9 and FGF23 recently have been implicated in postnatal cardiac growth. FGF23, a bone-derived hormone that regulates serum phosphate levels, has been found to cause pathological cardiac hypertrophy, an effect mediated by calcineurin-NFAT signaling in isolated rat cardiomyocytes [16]. Meanwhile, mice with inducible cardiomyocyte-specific expression of FGF9 developed physiological cardiac hypertrophy with normal diastolic and systolic function and no changes in the expression of pathological markers (*Anp*, *Bnp, βMHC*) [17]. Following MI, these mice developed increased hypertrophy, greater capillary density, decreased interstitial fibrosis, and attenuated induction of pathological markers compared to control mice [17]. This resultant physiological hypertrophy, however, was not the result of direct FGF signaling in the cardiomyocyte, but rather the result of endothelial cell-mediated paracrine signaling via BMP6 [17]. 

Despite a relative abundance of studies investigating FGF ligands in the development of cardiac hypertrophy, little is known about the role of FGF receptors in this process. In this study, we aimed to further understand the mechanisms governing FGF signaling in the development of cardiac hypertrophy, specifically with regard to how cardiomyocytes respond to a direct FGF signal via FGF receptor 1 (FGFR1). Given that overexpression of FGF2 alone is not sufficient to induce hypertrophy and that FGF9-mediated physiological hypertrophy is indirect, we hypothesized that adult cardiomyocytes may not be competent to transduce an FGF signal under baseline conditions, and that this repression would be relieved following injury or stress to the heart. 

**Figure 1:**
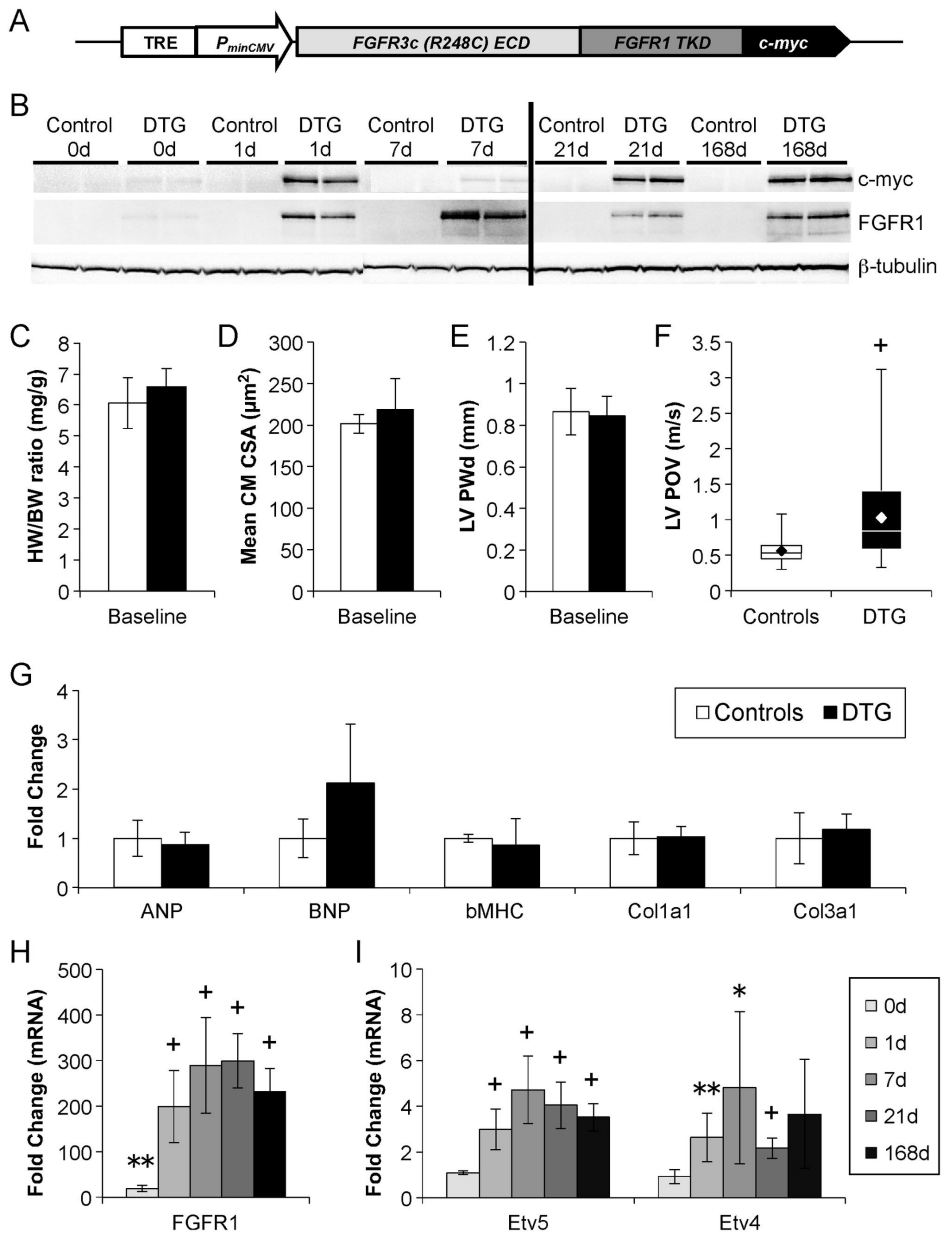
Inducible constitutively-active FGFR1 genetic system shows minimal baseline activity and rapid activation of transgene expression. (A) Schematic of a tetracycline response element (TRE)-*Fgfr31c*(R248C)*-*c-myc transgene. (B) Western blot analysis demonstrates minimal baseline expression (DTG 0d), rapid induction (DTG 1d), and consistent expression of caFGFR1 following feeding of DOX chow for various lengths of time (DTG 7d, 21d, 168d). ß-tubulin expression demonstrates equal protein loading. (C-E) Uninduced DTG mice show no evidence of hypertrophy on gross measurement of biventricular weight/body weight ratio (HW/BW, B; n_DTG_=7, n_control_=7), histological measurement of mean cardiomyocyte cross sectional area (CM CSA, C; n_DTG_=5, n_control_=5), or echocardiographic measurement of diastolic left ventricular posterior wall thickness (LVPWd, D; n_DTG_=47, n_control_=23). (F) Minimal expression of caFGFR1 is sufficient to induce a small, but significant, increase in outflow velocity from the proximal LV (n_DTG_=72, n_control_=43). (G) QRT-PCR analysis (n_DTG_=4, n_control_=4) shows no significant changes in markers of pathological cardiac remodeling. (H) QRT-PCR analysis (DTG: n_0d_=4, n_1d_=7, n_7d_=5, n_21d_=6, n_168d_=4) shows a significant elevation in Fgfr1 mRNA in uninduced DTG animals (0d) that dramatically increases within one day of transgene induction (1d). (I) No significant changes were seen in downstream transcription factors Etv5 or Etv4 at baseline (0d), but significant induction was observed after one day of DOX induction (1d). Fold change is versus corresponding time-matched controls (not shown; n_0d_=4, n_1d_=6, n_7d_=4, n_21d_=5, n_168d_=4). Error bars = standard deviation. *p<0.05, ** p<0.01, + p<0.001.

To directly test this model, we engineered a transgenic mouse line in which a ligand-independent constitutively-active FGF receptor (caFGFR1-myc) could be cell-autonomously induced specifically in cardiomyocytes through doxycycline (DOX) administration to α*MHC-rtTA, TRE-caFgfr1-myc* double transgenic mice (DTG, Figure 1A). We found that *in vivo* induction of caFGFR1 in adult cardiomyocytes led to rapid changes in cardiomyocyte contractile dynamics and a dynamic mid-cavity obstruction in the left ventricle. This acute pathology was followed by the progressive development of concentric hypertrophy with increased cardiac mass and cardiomyocyte size, interstitial fibrosis, and myocyte disarray characteristic of HCM. Interestingly, systolic function was preserved, even after six months of transgene induction. Examination of signaling pathways suggested a mechanism in which activation of FGF signaling in cardiomyocytes leads to the enhancement of intrinsic actin-myosin interactions, a common mechanism implicated in the pathogenesis of familial HCM. As such, this model of caFGFR1 overexpression-induced HCM may be useful for further investigation into the mechanisms involved in HCM development and studies aimed at its reversal.

## Materials and Methods

### Generation of TRE-caFGFR1 transgenic mice.

TRE-caFGFR1 mice were generated by subcloning a cDNA fragment encoding FGFR31C(R248C)-cmyc into pTRE2 (Invitrogen). The resultant pTRE2-FGFR31C(R248C) vector was linearized and injected into fertilized FVB oocytes. Eight founder lines were obtained and four were screened by crossing the founder to αMHC-rtTA transgenic mice, feeding double transgenic offspring doxycycline chow (Research Diets), and assaying for transgene expression by western blot. Western blotting was performed using antibodies against the c-myc epitope and β-tubulin (see more detailed description of Western blot below). All mice were maintained on a mixed background, primarily C57/Bl6/129. Both male and female mice were used in this study.

All procedures complied with the standards for the care and use of laboratory animals as stated in the *Guide or the Care and Use of Laboratory Animals* (NIH publication No. 85-23, revised 1996), and all protocols were approved by the Animal Studies Committee at Washington University School of Medicine.

### Echocardiography

Non-invasive ultrasound examination of the cardiovascular system was performed using a Vevo 2100 Ultrasound System (VisualSonics Inc, Toronto, Ontario, Canada) according to the following procedures. First, mice were lightly anesthetized with an intraperitoneal injection of 2% Avertin (tribromoethanol, 0.005 ml/g). One fifth of the initial dose was given as a maintenance dose at regular intervals depending on the level of anesthesia. Low-dose Avertin was used because it does not have significant negative inotropic or chronotropic effects. Hair was removed from the anterior chest with a combination of shaving and chemical hair remover, and the animals were placed on a warming pad in a left lateral decubitus position. Ultrasound coupling gel was applied to the chest. Examination of cardiac structure and function under these near-physiologic conditions was obtained with hand-held manipulation of the ultrasound transducer. Care was taken to maintain adequate contact while avoiding excessive pressure on the chest. Complete two-dimensional, M-mode, and Doppler ultrasound examination was performed from multiple views. After completion of the imaging studies, mice were kept warm and allowed to recover until returned to their cage. Digitally acquired images were analyzed off-line on a computer workstation running Vevo 2100 analysis software.

### Hemodynamic Analysis

Adult mice were anesthetized with thiopental sodium (60 mg/kg IP). This anesthesia produces a near physiologic heart rate of 500 beats/min, while still allowing for a surgical plane of anesthesia. The mice were intubated and ventilated with a Harvard ventilator set at 200-400 μl. The bilateral carotid arteries were identified in the region of the trachea and the right carotid was cannulated with a 1.4 French high fidelity micromanometer pressure-volume catheter (SciSense Advantage System, London, Ontario, Canada). The catheter was advanced retrogradely through the aortic valve into the left ventricle to assess pressure volume loops. Echocardiography was utilized when appropriate to ensure proper localization of the catheter and rule out catheter entrapment. Continuous aortic pressures, LV systolic and diastolic pressures, the derivative of LV pressure (dP/dT), and tau were recorded and analyzed with SciSense analysis software. Pressure-volume loops were analyzed before, during, and after an occlusion of the transverse aorta to assess load-independent measurements of myocardial contractility. 

### Exercise tolerance

To determine if chronic induction of caFGFR1 and the resulting concentric hypertrophy would result in impaired performance during forced exercise, adult mice were fed DOX chow for six months and then subjected to an acute treadmill stress test, where their exercise tolerance was measured by the length of time they were able to run unassisted (time to failure). Exercise tests were carried out using a state-of-the-art Exer4-OxyMax motorized treadmill (Columbus Instruments, Columbus, Ohio). Electrified bars delivered a very mild shock when a mouse failed to keep pace with the belt. Mice began running at 10 m/min, and the speed was increased by 3 m/min every 3 minutes. Time to failure was reached when a mouse remained on the electrified bar for a continuous 5 seconds. 

### Gross pathology and histological analysis

At study endpoints, mice were fully anesthetized with 2.5% Avertin (0.015 ml/g IP), and total body weight was measured. Hearts were removed, rinsed briefly in PBS, and then placed into a solution of PBS saturated with potassium chloride to arrest hearts in diastole. Both outflow tract and atria were carefully dissected away, and biventricular weight was measured. Normalized heart size was determined by calculating the ratio of biventricular weight (mg) to overall mouse weight (g; heart weight: body weight ratio). The apical third of the heart was removed and flash frozen in liquid nitrogen for protein or RNA analysis, while the remaining heart tissue was fixed in 10% neutral buffered formalin for 24-48 hours at room temperature. Samples were then dehydrated in 70% ethanol, embedded in paraffin, cut in 5 µm sections, and stained with H&E. Fibrosis was detected on sections using a Picrosirius red stain kit (24901, Polysciences, Inc., Warrington, PA) with Weigert’s iron hematolxylin counterstaining (HT1079, Sigma-Aldrich, St. Louis, MO). 

Fluorescein-tagged wheat germ agglutinin (FITC-WGA, L4895, Sigma-Aldrich, St. Louis, MO) was used to label all cell membranes, thus illuminating areas of increased interstitial cells and also making quantitation of cardiomyocyte cross-sectional area possible. Sections were dewaxed using xylenes, rehydrated through an ethanol series to water, rinsed in PBS, and then stained with FITC-WGA (1:100) for one hour at room temperature. Finally, sections were rinsed and mounted in Vectashield Hard Set with DAPI (H-1500, Vector Laboratories, Burlingame, CA), and imaged using a Zeiss Apotome Microscopy system. Five to ten images showing cardiomyocytes in cross section were taken from different regions of the ventricular wall in each sample, and mean cardiomyocyte cross sectional area was determined by tracing (using ImageJ software) the membrane of at least 300 cardiomyocytes per heart.

### Protein extraction and Western blotting

Protein was extracted from flash frozen apical pieces of the heart using RIPA buffer with freshly added 2% Protease Inhibitor Cocktail (P8340, Sigma-Aldrich) and Phosphatase Inhibitor Cocktail I and II (P2850 and P5726, Sigma-Aldrich). Protein concentration was determined utilizing a Pierce BCA assay kit (23225, Thermo Scientific, Rockford, IL). Total protein (60 µg) was separated on NuPAGE Novex 4-12% Bis-Tris Midi gels (WG1401, Invitrogen, Grand Island, NY) and transferred to PVDF membranes (iBlot Gel Transfer Stacks PVDF Regular, IB4010, Invitrogen). Membranes were blocked for one hour at room temperature with gentle shaking in TBST (50mM Tris, pH7.4, 150mM NaCl, 0.1% Tween20) containing 5% nonfat milk, and then probed with the antibodies listed in Table S2 in File S1 overnight at 4°C. After three rinses in TBST, membranes were incubated for one hour at room temperature in horseradish peroxidase-linked secondary antibodies, as described in Table S2 in File S1, in TBST with 5% nonfat milk, rinsed again in TBST, and developed using SuperSignal West Femto Maximum Sensitivity Substrate (34096, Thermo Scientific).

The membrane was then stripped utilizing multiple washes of boiled 100 mM glycine and TBST wash buffer at room temperature (glycine reprobing protocol using the iBlot dry blotting system, Invitrogen) and sequentially reprobed with additional antibodies. Protein bands were quantified by densitometry, and protein loading was normalized to β-tubulin. Phospho-ERK1/2 and phospho-Akt were normalized to total-ERK1/2 and total-Akt protein levels, and then scaled relative to controls, where control samples were set at a value of 1.

### RNA isolation, cDNA synthesis, and quantitative RT-PCR

RNA was extracted from flash frozen apical pieces of the heart using a Qiagen RNeasy Mini kit (74104, Qiagen, Valencia, CA), with on-column DNA digest. RNA concentration was determined utilizing a Nanodrop spectrophotometer. cDNA was made using the BioRad iScript Reverse Transcription Supermix for RT-qPCR kit (170-8841, BioRad, Hercules, CA). Quantitative RT-PCR was performed on an Applied Biosystems (ABI) 7500 thermocycler using ABI Taqman® Fast Advaned Master Mix (ABI #4444557) and Taqman® gene expression assays listed in Table S3 in File S1 (ABI). All samples were normalized to *Hprt* and then scaled relative to controls using the standard ddCt method.

### Inhibitor studies

Eight- to 12-week-old MHC-rtTA, TRE-caFGFR1 mice were treated with one of the following drugs, starting one week prior to addition of DOX chow and continuing throughout the one week of transgene induction: Losartan (600mg/L in drinking water, Sigma #61188), propranolol (500mg/L in drinking water, Sigma #P0884), diltiazem (450mg/L in drinking water, Sigma #D2521), Cyclosporine A (15mg/kg twice daily subcutaneous injections, Sigma #30024), Wortmannin (1mg/kg/d IP, Sigma #W1628), U0126 (1mg/kg IP every 3 days, Calbiochem #662005), or PD173074 (1mg/kg/d IP, Pfizer), according to published studies [18–23].

### Myocyte Isolation, cell contractility, and sarcomere length measurements

Ventricular myocytes from acutely-induced (24-48 hours following doxycycline treatment) control or double transgenic (caFGFR1-expressing) mice were isolated as previously described [24]. Following isolation, the ventricular myocyte suspension was washed 3 times in Wittenberg Isolation medium containing (in mM): 116 NaCl, 5.4 KCl, 8 MgCl_2_, 1 NaH_2_PO_4_, 1.5KH_2_PO_4_, 4 NaHCO_3_, 12 glucose, 21 *N*-(2-hydroxyethyl) piperazine-N’-(2-ethanesulfonic acid) (HEPES), 2 glutamine, supplemented with 1X essential vitamins (GIBCO), 1X essential amino acids (GIBCO), 50mg/ml BSA, 12.5mg/ml Taurine, and with increasing concentrations of CaCl_2_ (150µM, 400µM, and 900µM). The cells were then washed with Tyrode’s solution containing (in mM) NaCl, 137; KCl, 5.4; NaH_2_PO_4_, 0.16; glucose, 10; CaCl_2_, 1.0; MgCl_2,_ 0.5; HEPES, 5.0; NaHCO_3_, 3.0; pH 7.3–7.4. Freshly isolated myocytes were transferred to a recording chamber mounted on the stage of a Nikon Diaphot inverted microscope and superfused at 60ml/hour with normal Tyrode’s solution. The cells were allowed to settle for 5 minutes and were subsequently paced (5-10V) at 0.5 Hz for 5 minutes before recording. All experiments were performed at room temperature. Video images of individual myocyte contractions were acquired using a Myocam camera (IonOptix), and a Fourier transform was applied to the dark/light contrast of the A and I bands to calculate the sarcomere length. Length traces were subsequently analyzed with the Ion Wizard software (IonOptix). To assess the relaxation of diastolic sarcomere length, cells were loaded with 100µM BAPTA-AM (Sigma) for 1 hour prior to recording or were treated with Tyrode’s solution containing 40mM 2, 3-butanedione monoxime (BDM) (Sigma) during recording.

### Statistical analysis

In all figures, error bars represent standard deviation, unless otherwise noted. Statistical analysis was carried out using a standard two-tailed student T-test, with a p<0.05 considered significant. Individual outliers were determined using Grubbs’ test.

## Results

### Doxycycline-inducible cardiomyocyte-specific constitutively-active FGF receptor 1 mouse model

To investigate the cell autonomous function of FGF signaling in adult cardiomyocytes, we utilized the doxycycline (DOX)-regulatable TET-on system with the *αMHC-rtTA* mouse line [25]. We generated a tetracycline response element (TRE)-*Fgfr31c*(*R248C*)*-c-myc* transgene, in which FGFR31c(R248C) is a chimeric receptor consisting of the FGFR3c(R248C) mutant extracellular and transmembrane domains fused to the FGFR1 tyrosine kinase domain (Figure 1A). The FGFR3c(R248C) mutation confers ligand-independent constitutive activity [26]. By fusing this mutant FGFR3c receptor to the FGFR1 tyrosine kinase domain, one can produce a receptor that constitutively activates FGFR1 signaling in the absence of ligand. A c-myc tag was placed at the C-terminal tail of FGFR1 to allow detection of mutant receptor expression. For simplicity, the *TRE-Fgfr31c*(*R248C*)*-c-myc* transgene will be referred to as *TRE-caFgfr1*.

*αMHC-rtTA/TRE-caFgfr1* double transgenic (DTG) mice were examined for expression of the c-myc tag and FGFR1 in the absence of DOX to determine the basal level of transgene activation and native FGFR1 expression in the adult mouse heart. DTG mice without DOX displayed very low levels of c-myc and FGFR1 expression (Figure 1B, DTG 0d), while single transgenic TRE-caFGFR1 animals did not express the transgene or native FGFR1 (Figure 1B, control 0d). To determine if this level of basal transgene expression led to a cardiac phenotype, we measured the ratio of biventricular heart weight and total body weight (HW/BW ratio, Figure 1C), mean cardiomyocyte cross-sectional area (Figure 1D), and in vivo left ventricle (LV) diastolic posterior wall thickness (LVPWd, Figure 1E) and proximal LV peak outflow velocity (LV POV, Figure 1F) in 12- to 14-week-old mice. Cardiac mass, myocyte size, and LV wall thickness were comparable between DTG mice and controls, while a small, but significant, increase was observed in LV POV (1.01±0.61m/s vs. 0.57±0.18m/s, p<0.001). 

We also utilized quantitative RT-PCR (qRT-PCR) to examine the expression of markers of heart failure, hypertrophy, and fibrosis (Figure 1G) [5,6,27,28]. While the expression of b-type natriuretic peptide (Bnp) was slightly elevated, levels of atrial natriuretic peptide (Anp), beta-myosin heavy chain (*ßmhc*), and collagens 1 and 3 (*Col1a1*, *Col3a1*) were unchanged in uninduced DTG hearts. In all studies, littermate controls included single transgenic mice (*αMHC-rtTA* only, *TRE-caFgfr1* only) or wild-type mice. No significant differences were observed between the three control genotypes in any of the analyzed parameters, and as a result, control data were pooled.

To test whether αMHC-rtTA can confer inducible expression of caFGFR1 in the heart, we examined c-myc and FGFR1 expression following the administration of DOX chow. One day of DOX was sufficient to induce robust transgene expression, which was maintained at consistent levels with chronic feeding of DOX chow (Figure 1B). No transgene activation or native FGFR1 expression was visible in controls. We also utilized qRT-PCR to measure changes in *Fgfr1* gene expression. While *Fgfr1* mRNA expression was significantly increased at baseline (0d) in DTG mice (19.6±6.6-fold, p=0.001), expression increased dramatically after one day on DOX (199±80-fold, p<0.001) and was maintained at these levels with chronic DOX feeding (Figure 1H). Additionally, we monitored gene expression of the FGF-responsive ETS transcription factors *Etv4* and *Etv5* (Figure 1I) [29,30]. Despite increased *Fgfr1* expression, *Etv4* and *Etv5* gene expression remained unchanged at baseline in DTG mice compared to controls. Expression of both transcription factors increased significantly after one day of transgene induction (*Etv5*:3.0±0.9-fold, p<0.001; *Etv4*:2.6±1.1, p=0.004), and remained elevated with chronic expression of caFGFR1.

### In vivo characterization of phenotype development in *αMHC-rtTA, TRE-caFgfr1* mice

**Figure 2:**
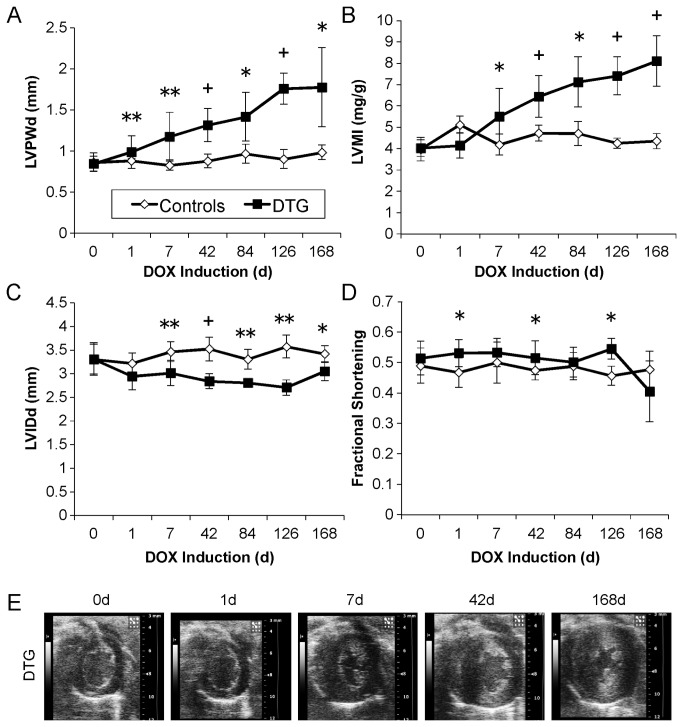
Induction of caFGFR1 in adult cardiomyocytes results in the development of concentric hypertrophy with preservation of systolic function over time. (A) LV diastolic posterior wall thickness (LVPWd, A) was significantly elevated by one day of induction, while LV mass index (LVMI) was significantly elevated (B) and LV internal diastolic diameter (LVIDd) was significantly decreased (C) within seven days of DOX induction. (D) Systolic function was maintained throughout 6 months of transgene induction. Error bars = standard deviation. *p<0.05, **p<0.01, +p<0.001. 0d: n_DTG_=47, n_control_=23; 1d: n_DTG_=8, n_control_=4; 7d: n_DTG_=17, n_control_=7; 42d: n_DTG_=14, n_control_=12; 84d: n_DTG_=4, n_control_=4; 126d: n_DTG_=4, n_control_=4; 168d: n_DTG_=4, n_control_=4. (E) Representative short axis echocardiographic images depicting the progression of hypertrophy in DTG animals from baseline to 168 days of induction.

To determine the effect of activating FGF signaling in cardiomyocytes, 12- to 14-week-old DTG animals and controls were induced, and cardiac function was monitored over time with echocardiography. LV diastolic posterior wall thickness (LVPWd) was significantly elevated in DTG mice within one day of DOX induction (0.99±0.20 vs. 0.88±0.02mm, p=0.001, Figure 2A) and LV mass index was significantly elevated by one week of induction (5.3±1.0 vs. 4.2±0.5mg/g, p=0.02, LVMI, Figure 2B), and both parameters continued to increase throughout six months of induction. LV internal diastolic diameter (LVIDd) was significantly decreased within one week of transgene induction (3.04±0.26 vs. 3.47±0.21mm, p=0.001, Figure 2C) and remained relatively constant over time. Systolic function, as measured by fractional shortening (FS) (Figure 2D), remained comparable between DTG animals and controls, and was even significantly increased at three time points (1d, 42d, 126d). Video S1 provides representative short axis echocardiography showing cardiac function and progression of hypertrophy from baseline (0d DTG) through six months (168d DTG) of induction, while Figure 2E displays representative diastolic mid-ventricle short-axis still images from DTG animals. Note the concentric and symmetrical increase in wall thickness, the decreased chamber volume, and the apparent increase in cardiac contractility. 

**Figure 3:**
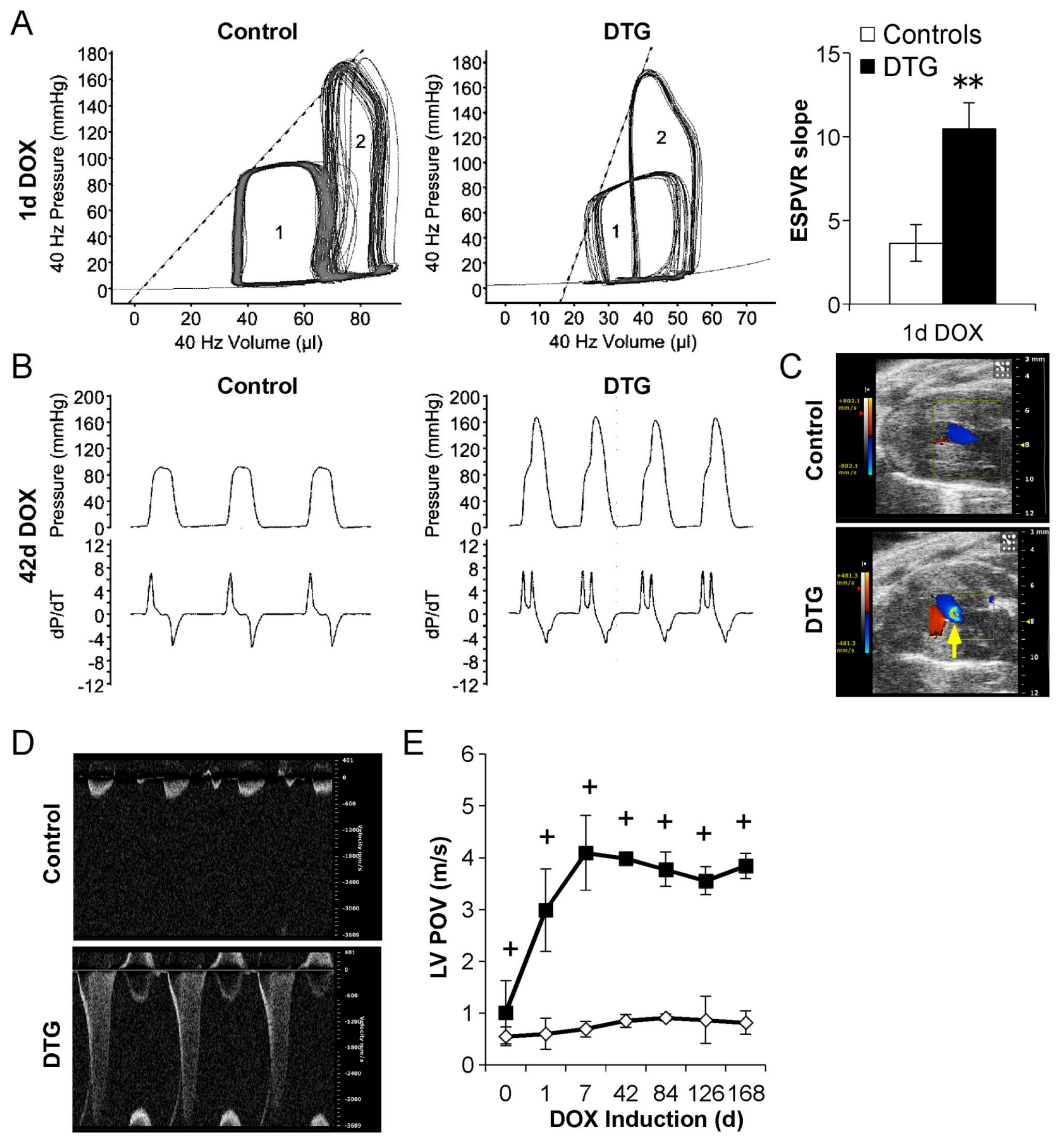
In vivo induction of caFGFR1 in adult cardiomyocytes leads to a hypercontractile phenotype with the development of a dynamic obstruction in the proximal left ventricle. (A) Representative pressure-volume (PV) loops at baseline (1) and following increased afterload (2) in DTG mice and littermate controls induced with DOX chow for 24 hours. The slope of the end systolic pressure-volume relationship (ESPVR, dotted line) is significantly elevated in DTG mice (n=3 for DTG and control). (B) Representative pressure (top) and dP/dt (bottom) tracings suggesting the presence of a dynamic obstruction in DTG mice induced for 42 days (n_DTG_=3, n_control_=2). (C) Representative color Doppler images (blue = blood outflow, red = blood inflow) and (D) spectral Doppler tracings illustrating flow convergence (yellow arrow) and a high velocity late-peaking jet originating from the point of dynamic mid-cavity obstruction in DTG mice. (E) Quantitation of LV peak outflow velocity (LV POV) at the point of flow convergence. Error bars = standard deviation. *p<0.05, **p<0.01, +p<0.001.

To determine the effects of caFGFR1 on the relaxation and contractile properties of the adult heart, we performed an in vivo hemodynamic assessment after one day (Figure 3A, Table S1 in File S1) and six weeks (Figure 3B) of transgene induction. Following one day of induction, no significant differences were observed in heart rate, dP/dt_max_, dP/dt_min_, end systolic or diastolic pressure (ESP, EDP), or tau (Glantz) values of relaxation (Table S1 in File S1). However, end systolic volume (ESV) was greatly reduced (18.2±3.3 vs. 31.7±4.5mL, p=0.05), and end diastolic volume (EDV) was significantly decreased in DTG mice compared to controls (31.8±2.8 vs. 51.2±4.5mL, p=0.01). To assess load-independent measures of contractility, the LV pressure-volume (PV) relationship was assessed by briefly constricting the transverse aorta and determining the end-systolic pressure-volume relationship (ESPVR) between the baseline and increased afterload PV loops. The slope of the ESPVR was significantly elevated following 24 hours of caFGFR1 induction (10.4±1.6 vs. 3.6±1.1, p=0.004, Figure 3A), suggestive of increased contractility [31].

Six weeks of transgene induction resulted in an obstructive phenotype, as indicated by the altered appearance of the LV pressure recordings and dP/dt tracings seen in DTG animals compared to controls (Figure 3B). Echocardiography was utilized to ensure proper catheter placement and rule out catheter entrapment in the ventricular wall as a cause of the altered pressure tracings. The presence of marked dynamic outflow obstruction and consequent alteration of PV loops precluded reliable analysis of ESPVR after prolonged transgene induction.

Echocardiographic Doppler analysis supported the findings from the hemodynamic studies. Representative color Doppler images from DTG mice induced for six weeks (Figure 3C, bottom) showed a distinct point of flow convergence in the mid-LV that was not seen in control mice (Figure 3C, top). Spectral Doppler interrogation revealed markedly elevated late-peaking outflow velocities at the point of the flow convergence (Figure 3D,E). As stated previously, DTG mice showed elevated outflow velocities at baseline (Figure 1F); however, these values were substantially lower than velocities seen after 24 hours of transgene induction (0d:1.01±0.61 vs. 1d:2.98±0.80m/s, p<0.001) and did not affect phenotype development. However, where possible, mice with peak outflow velocities greater than 1.0 m/s at baseline were not utilized for further analysis.

### Histologic characterization of phenotype and pathologic gene expression analysis

**Figure 4:**
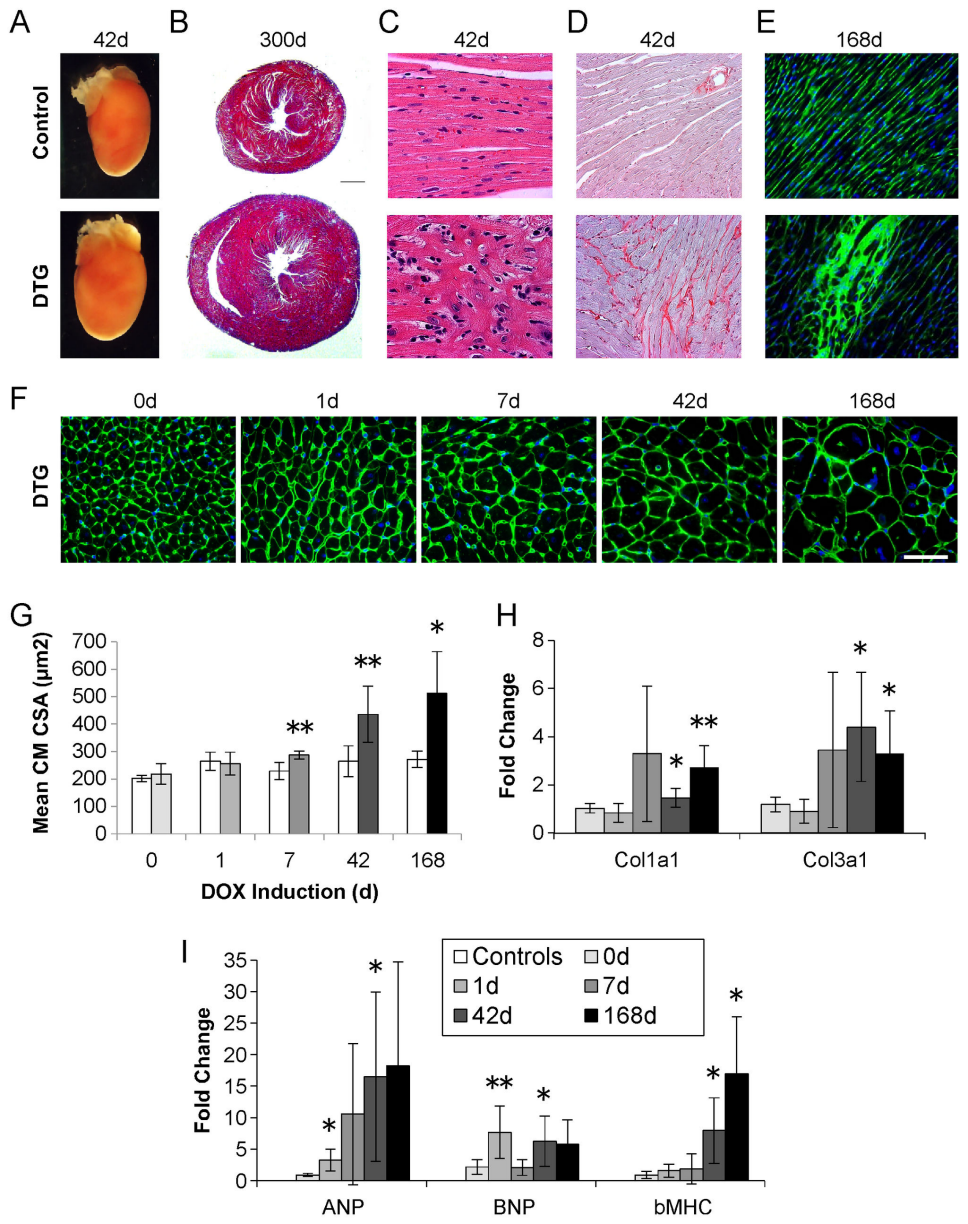
Chronic induction of caFGFR1 results in a pathologic state with molecular and histologic characteristics of hypertrophic cardiomyopathy. (A) Induction of caFGFR1 for 42 days results in grossly enlarged hearts in DTG compared to control mice. (B) Following 300 days of transgene induction, massive DTG hearts show concentric hypertrophy with no evidence of progression to dilatation (scale bar = 1mm). (C,D) Histological examination of DTG hearts induced for 42 days reveals patchy areas of myocyte disarray (H&E, 20x magnification, C) and fibrosis (picrosirius red, 20x magnification, D). Six months (168d) of transgene induction results in patchy areas of increased interstitial cells illustrated by fluorescein-tagged wheat germ agglutinin staining (FITC-WGA, 40x magnification, E). (F) FITC-WGA staining illustrating the progressive enlargement of cardiomyocytes in DTG mice (scale bar = 20mm). (G) Quantitation of mean cardiomyocyte cross-sectional area (CM CSA). (H,I) QRT-PCR reveals significant upregulation of pathological markers of LV remodeling. Fold change is versus corresponding time-matched controls (not shown). DTG: n_0d_=4, n_1d_=7, n_7d_=5, n_42d_=4, n_168d_=4; controls: n_0d_=4, n_1d_=6, n_7d_=4, n_42d_=5, n_168d_=4. Error bars = standard deviation. *p<0.05, **p<0.01.

To further characterize the hypertrophy phenotype in DTG mice, we performed gross and histological analysis of hearts from DTG and control mice. Following 42 days of caFGFR1 induction, hearts were visibly larger in DTG animals (Figure 4A). This difference was more apparent after 300 days of transgene expression, evident in Trichrome-stained cardiac cross-sections (Figure 4B). After ten months of caFGFR1 expression, concentric hypertrophy was still present, without any progression to LV dilatation. Patchy areas of myocyte disarray (Figure 4C) and increased interstitial fibrosis (Figure 4D) were observed after 42 days of induction. Wheat germ agglutinin staining showed large patches of densely packed interstitial cells after six months of induction (Figure 4E) and progressively increasing myocyte size with transgene induction (Figure 4F). Cardiomyocyte cross-sectional area was significantly increased by one week of caFGFR1 induction (288±15 vs. 229±32µm^2^, p=0.002) and continued to increase through six months of induction (512±152 vs. 271±30µm^2^, p=0.02, Figure 4G). 

To confirm that the hypertrophy seen in DTG mice was pathological, qRT-PCR was used to examine the expression of markers of pathological ventricular remodeling (Figure 4H & I). *Anp* and *Bnp* were significantly elevated in DTG mice after one day of caFGFR1 induction (*Anp*:3.2±1.7-fold, p=0.01; *Bnp*:7.7±4.2-fold, p=0.003), while *ßMHC, Col1a1*, and *Col3a1* were not significantly elevated until 42 days of transgene induction (*ßMHC*:7.9±5.2-fold, p=0.02; *Col1a1*:1.5±0.4-fold, p=0.04; *Col3a1*:4.4±2.3-fold, p=0.01).

### Exercise tolerance

Double transgenic mice expressing caFGFR1 exhibited all of the pathophysiological characteristics of hypertrophic cardiomyopathy (HCM). Because one of the most common clinical manifestations of HCM is exercise intolerance [3], it was important to determine if chronic induction of caFGFR1 and the resulting concentric hypertrophy would result in impaired performance during forced exercise. Adult mice were fed DOX chow for six months and then subjected to an acute treadmill stress test, where their exercise tolerance was measured by the length of time they were able to run unassisted (time to failure). Interestingly, caFGFR1-expressing double transgenic mice did not display any signs of exercise intolerance and were capable of running for the same length of time as their littermate controls (24.4 ± 1.0 vs. 21.5 ± 4.7 minutes, Figure S1 in File S1).

### Reversibility of phenotype

**Figure 5:**
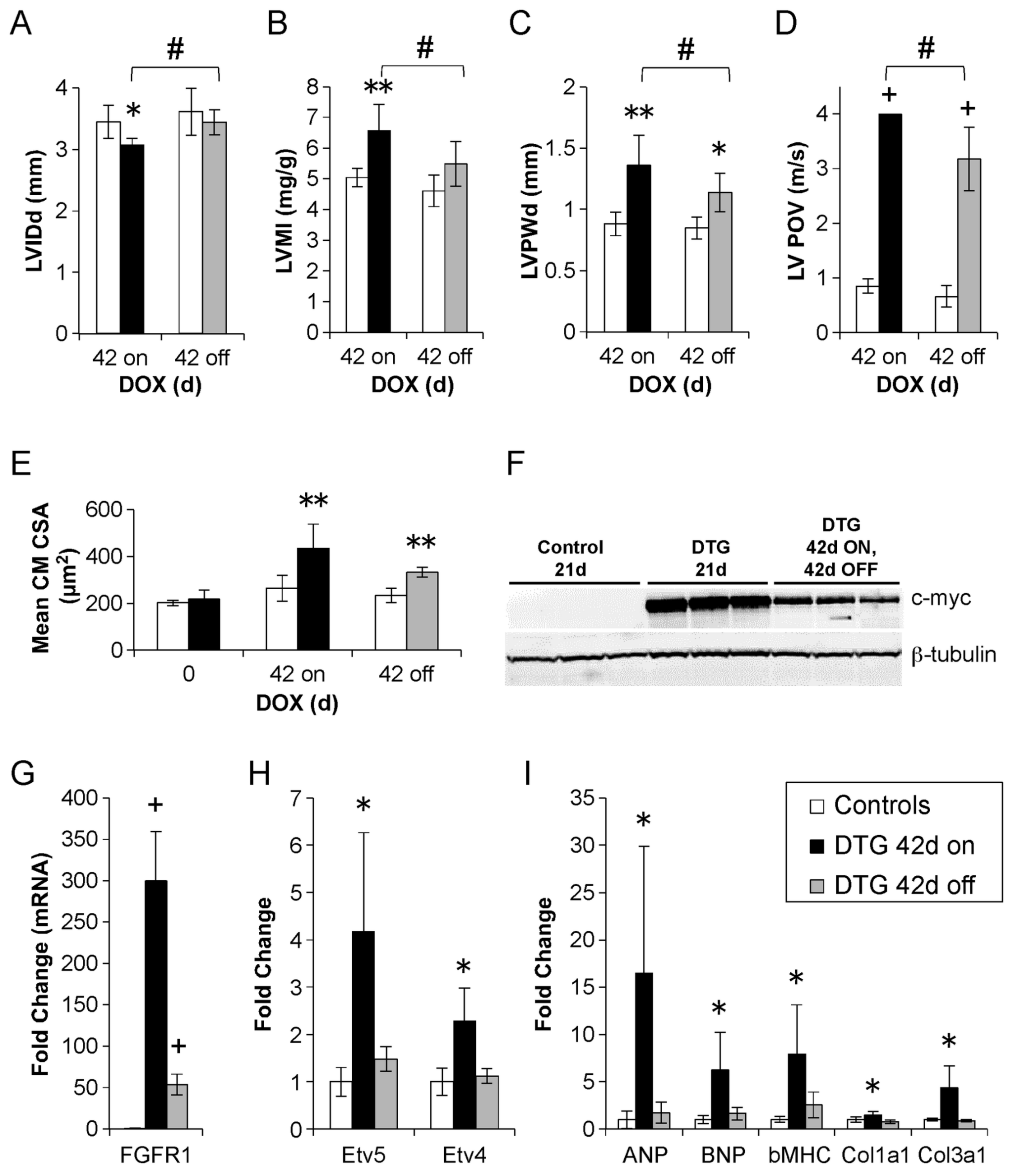
Transgene induction for 42 days, followed by DOX removal for 42 days leads to partial phenotype reversal. (A,B) LV internal diastolic diameter (LVIDd, A) and LV mass index (LVMI, B) returned to near control levels after removal of DOX for 42 days (42 off). (C) LV diastolic posterior wall thickness (LVPWd) was significantly reduced in DTG mice (n=6) following DOX removal (42 off), but remained significantly elevated compared to controls (n=4). (D) Despite decreasing significantly following removal of DOX, peak outflow velocity (LV POV) from the proximal ventricle was still significantly elevated compared to controls. (E) Cardiomyocyte cross-sectional area (CM CSA) was smaller than that seen following six weeks of caFGFR1 induction (42 on), but cells were still significantly larger than controls following DOX removal (42 off). (F) Western blot analysis showing a significant decrease but persistent expression of caFGFR1 following removal of DOX (DTG 42d ON 42d OFF), compared to transgene expression after three weeks of induction (DTG 21d; protein samples from six weeks of induction were unavailable). (G-I) QRT-PCR analysis demonstrating continued overexpression of Fgfr1 in DTG hearts following 42 days off DOX (gray bar), despite a large reduction from the level of expression observed following 42 days on DOX (black bar, G). Expression of downstream mediators of FGF signaling (H) and markers of pathological LV remodeling (I) are decreased to control levels 42 days following DOX removal. Error bars = standard deviation. *p<0.05, **p<0.01, +p<0.001 compared to controls. #p<0.05 compared to DTG mice 42 days on DOX.

One of the advantages of the Tet-on system is its potential for reversibility—the removal of DOX inactivates the rtTA and halts transcription of the transgene. To assess the reversibility of the caFGFR1-induced phenotype, 12- to 14-week old DTG mice and littermate controls were induced with DOX chow for six weeks and then fed normal chow for six weeks. LV mass index and LV internal diastolic diameter returned to near control levels after DOX removal (Figure 5A,B). LV diastolic posterior wall thickness (Figure 5C) was significantly reduced by 16% after DOX removal, but remained significantly elevated over controls. LV peak outflow velocity (Figure 5D), measured at the point of flow convergence in the mid-ventricle, was the most resistant to reversal, decreasing by 20% after DOX removal, but remaining significantly elevated compared to controls. Consistent with in vivo wall thickness, cardiomyocyte cross-sectional area was reduced by 24% after DOX removal but remained significantly larger than controls (Figure 5E). 

To determine whether the continued presence of phenotype was the result of resistance to reversibility or continued expression of caFGFR1, Western blot analysis of c-myc expression and qRT-PCR analysis for *Fgfr1* gene expression were performed. caFGFR1, as measured by the c-myc tag, was still expressed at relatively high levels after 42 days of DOX removal (DTG 42d ON,42d OFF), although expression was significantly reduced compared to expression following three weeks of DOX induction (DTG 21d; Figure 5F). This continued expression of the c-myc tag following DOX removal corresponded to a 54-fold increase of *Fgfr1* compared to controls (p<0.001), down 82% from the 299-fold seen following transgene induction for 42 days (Figure 5G). Despite continued transgene expression, expression of downstream mediators of FGF signaling, *Etv5* and *Etv4* (Figure 5H), and markers of pathological ventricular remodeling (Figure 5I) returned to normal levels following 42 days off DOX.

### Mechanisms mediating phenotype development following induction of caFGFR1

FGF receptors can activate a variety of pathways, including MAPK/ERK, PI3K/Akt, PLCγ, and STATs [32]. In an effort to elucidate the mechanisms involved in the development of the hypercontractility and hypertrophy seen in DTG mice, we analyzed protein expression throughout the time course of caFGFR1 induction. While there was a trend towards a transient 2-fold increase in ERK1/2 activation after one day of induction, this change was not consistent enough to achieve significance (n_DTG_=8, n_control_=5, Figure S2 in File S1), and no changes were apparent beyond 24 hours of caFGFR1 induction. We also examined the activation of other MAPKs (p38, JNK), as well as Akt, PLCγ, and STATs (Stat3, Stat5), none of which showed any trends toward activation following induction of FGF signaling in cardiomyocytes (Figure S2 in File S1). 

**Figure 6:**
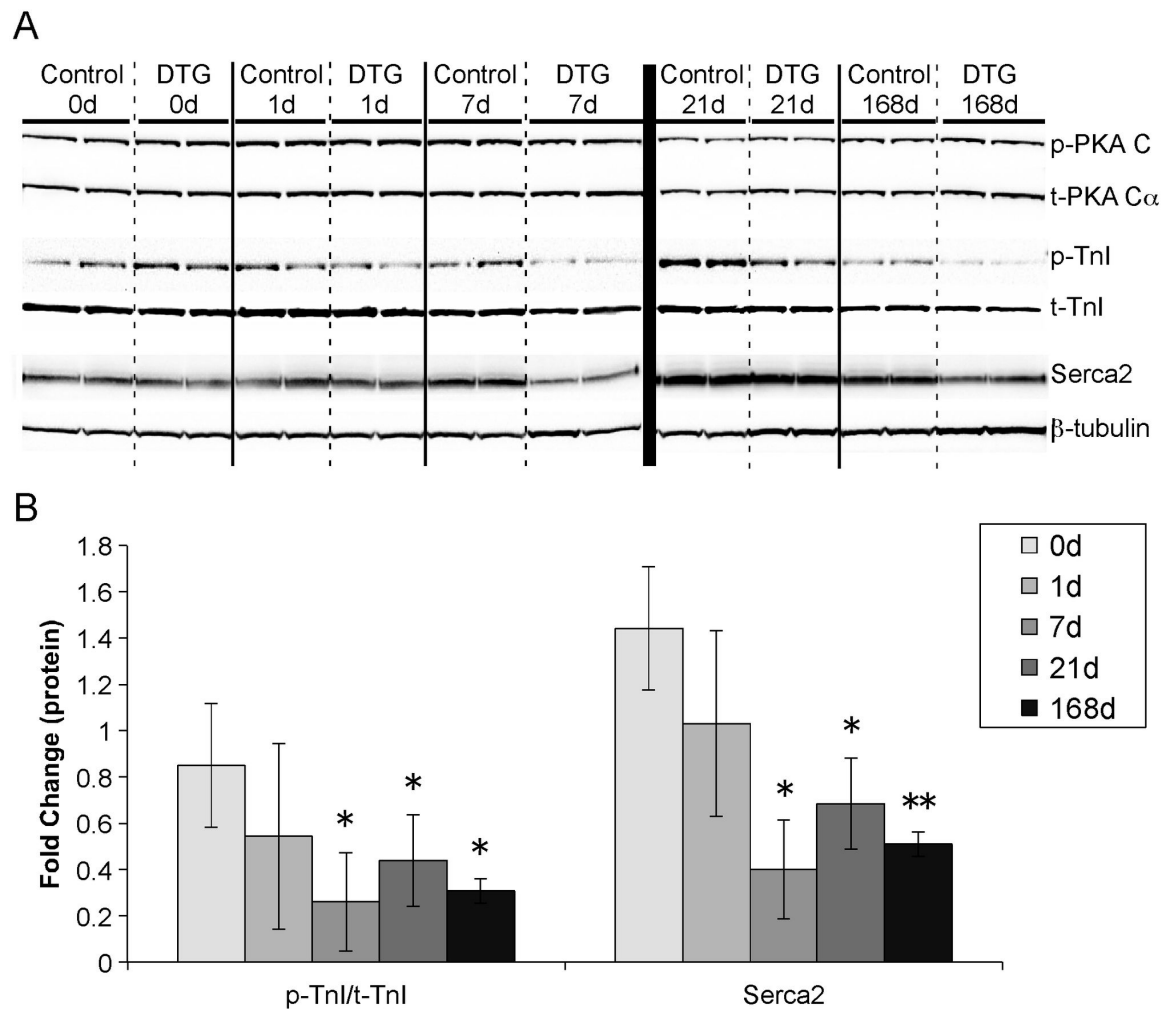
DTG mice have significantly decreased troponin I phosphorylation and Serca2 expression. (A) Representative Western blots depicting no changes in the expression of phosphorylated protein kinase A (P-PKA) and decreased expression of phosphorylated troponin I (P-TnI) and Serca2. ß-tubulin was used as a loading control. Independent blots are separated by a thick black line. (B) Densitometric quantitation of p-TnI/t-TnI and Serca2, demonstrating a significant decrease in both by seven days of caFGFR1 induction. Fold change is versus corresponding time-matched controls (not shown). DTG (shown): n_0d_=5, n_1d_=8, n_7d_=8, n_21d_=6, n_168d_=2; controls (not shown): n_0d_=2, n_1d_=5, n_7d_=4, n_21d_=3, n_168d_=2. Error bars = standard deviation. *p<0.05, **p<0.01.

Because there was no clear activation of a pathway, or pathways, directly downstream of the FGF receptor, we examined other signaling pathways that could result in hypertrophy or hypercontractility. We hypothesized that induction of caFGFR1 could lead to increased sensitization of cardiomyocytes to sympathetic signaling, since adrenergic activation in the heart has been shown to both increase contractility and cause hypertrophy in cardiomyocytes [33]. We examined downstream targets of adrenergic receptors, including protein kinase A (PKA), troponin I (TnI), and phospholamban. No changes were observed in PKA activation at any time during caFGFR1 induction. Phosphorylation of TnI was significantly decreased by one week of transgene expression (Figure 6), in contrast to what we would expect if adrenergic signaling were increased. There was also a trend towards decreased phosphorylation of phospholamban, but this result was more variable than TnI phosphorylation (data not shown). Alterations in calcium handling are also a common mechanism in the development of hypertrophy [3,4]. Consistent with a potential role for aberrant calcium signaling, we observed significant downregulation of Serca2 after seven days of transgene induction (Figure 6). In addition, ryanodine receptor (RyR2) expression was significantly decreased at both one day (57.6±4.8% of control, p<0.01) and seven days (56.2±3.1% of control, p<0.01) following transgene induction; whereas, there was no significant change in expression levels of the sodium-calcium exchanger (NCX1) at these time points (data not shown). 

### Cell autonomous effects of caFGFR1 expression on cardiomyocyte physiology

**Figure 7:**
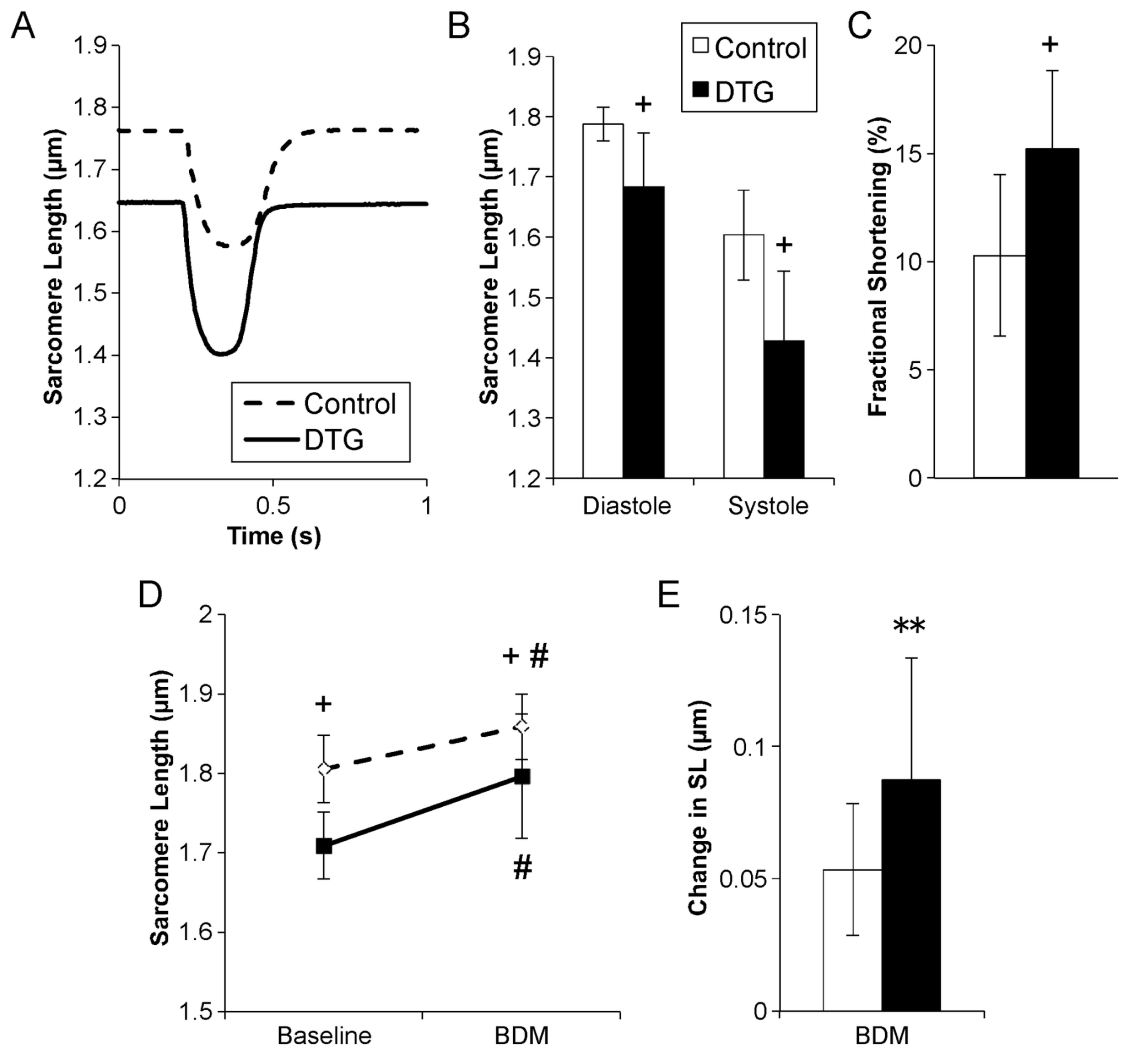
Ventricular myocytes from double transgenic mice have impaired relaxation and enhanced contraction. (A) Representative signal averaged sarcomere length measurements during contraction recorded from acutely-induced DTG (solid line) and control cardiomyocytes (dashed line). (B) DTG myocytes had significantly shorter sarcomere lengths at both systole and diastole, (C) with an overall increase in fractional shortening (n=4 mice, 17 myocytes total for both control and DTG). (D) Addition of BDM caused significant relaxation of both DTG and control myocytes, and (E) resulted in a greater change in sarcomere length in DTG myocytes (n=3 mice each, 19 control myocytes, 23 DTG myocytes). Error bars = standard deviation. **p<0.01, +p<0.001 DTG vs. controls; # p<0.05 vs. respective baseline measurements.

Ten- to twelve-week-old DTG and control mice were induced with doxycycline for 24-48 hours, and then unloaded cell contraction was assessed in acutely isolated ventricular myocytes. Representative traces of sarcomere length during myocyte contraction (Figure 7A) and quantitation of sarcomere length demonstrate that DTG myocytes had significantly shorter lengths at both diastole and systole (Figure 7B), with an overall increase in fractional shortening (Figure 7C) compared to myocytes from controls. These data suggest that caFGFR1 leads to both impaired relaxation and enhanced contraction in cardiomyocytes.

To further examine the basis of incomplete relaxation, 2,3-butanedione monoxime (BDM), which inhibits actin-myosin interactions and slows cross bridge cycling [34,35], was applied to isolated ventricular myocytes. BDM led to significant lengthening of both control and DTG sarcomere lengths (Figure 7D). More importantly, myocytes from DTG mice experienced a significantly greater increase in sarcomere length than controls and approached a similar final relaxed sarcomere length to controls following BDM addition (Figure 7E).

To determine whether the altered contractile properties in DTG myocytes were the result of increased intracellular calcium concentration (due to decreased calcium recycling), myocytes were loaded with BAPTA-AM, a membrane-permeant calcium chelator [35]. In contrast to BDM, there was no change in final relaxed sarcomere length in either control or DTG myocytes, suggesting that enhanced contractility is independent of cytosolic calcium levels (data not shown).

### Clinical usefulness

Two classes of drugs that have been proven to reverse pathological remodeling and improve cardiac function in heart failure are angiotensin receptor antagonists, such as losartan, and ß-adrenergic receptor antagonists, such as propranolol [36]. While the precise mechanisms by which beta-blockers improve cardiac function and promote reverse remodeling are unknown, this treatment is one of the mainstays for the symptomatic relief of obstructive HCM due to its ability to decrease the outflow gradient [36,37]. Meanwhile, losartan is contraindicated in patients with LV outflow obstruction due to its effect on blood pressure (which can increase the outflow gradient) [38], but recent studies have indicated that blockade of angiotensin signaling can prevent much of the pathological remodeling in animal models of HCM [23].

To examine the clinical usefulness of this new model of HCM, DTG mice were given losartan (80 mg/kg/d) or propranolol (67 mg/kg/d) in their drinking water to determine if these drugs could prevent or lessen phenotype development (Figures S3, S4 in File S1). Treatment began one week prior to the start of DOX chow, and continued throughout one week of transgene induction. Both drugs abrogated the hypertrophic response seen in untreated DTG mice induced for one week, as assessed by in vivo LV mass index (Figures S3A, S4A in File S1). The increase in LV posterior wall thickness was also moderately blocked with both drugs, although neither treatment caused a significant reduction (Figures S3D, S4D in File S1). Only propranolol significantly reduced the dynamic obstruction, as measured by LV outflow velocity (Figures S3B, S4B in File S1), although it was still significantly elevated compared to measurements taken prior to drug treatment and caFGFR1 induction (DTG+Pro, 0d) (Figure S3B in File S1). These studies suggest that this model of caFGFR1-induced HCM may prove useful to evaluate other treatments aimed at preventing or reversing the course of this pathological condition.

## Discussion

### Induction of caFGFR1 in adult cardiomyocytes results in hypercontractility and a hypertrophic cardiomyopathy

In this study, we created a new mouse model in which we could induce FGF receptor signaling specifically in cardiomyocytes in an attempt to better understand the relationship between cardiomyocyte FGF signaling and the development of hypertrophy. We hypothesized that adult cardiomyocytes are not competent to respond to an FGF signal due to repression of the FGF signaling pathway under homeostatic conditions. However, we discovered that cell autonomous activation of FGF signaling was sufficient to induce significant concentric hypertrophy within seven days of transgene expression that progressively increased throughout ten months of caFGFR1 expression. Patchy areas of fibrosis, elevated expression of pathological cardiac markers, myocyte disarray, and maintenance or enhancement of systolic function support the notion that sustained activation of FGF signaling in adult cardiomyocytes leads to a hypertrophic cardiomyopathy.

In addition to the development of HCM, caFGFR1 rapidly increases the contractility of the heart within 24 hours of induction. This hypercontractility resulted in narrowing of the ventricular cavity during systole, leading to a high velocity jet of blood flowing past the papillary muscles in the mid-ventricle. This effect was further amplified to the point of a dynamic obstruction following the development of concentric hypertrophy, as evidenced by the significant elevation in end systolic pressure in the distal ventricle. The hypercontractility phenotype is extremely sensitive to activation of FGF signaling, as it was significantly elevated even at baseline due to a small amount of caFGFR1 expression in DTG mice, and it was resistant to reversal following removal of DOX. Hypertrophic parameters approached near control levels after six weeks off DOX, while peak outflow velocity remained significantly elevated. The fact that DTG mice at baseline do not show signs of hypertrophy, despite having increased contractility, suggests that a low level of activated FGFR1 in cardiomyocytes directly regulates cardiac contractility, while prolonged exposure to high levels of activated FGFR1 leads to the development of HCM. 

### Constitutively-active FGFR1 in cardiomyocytes alters mechanisms common to HCM pathophysiology

HCM affects one out of 500 adults and is the most common cardiovascular genetic disorder. The majority of cases (45-70%) can be attributed to dominant mutations in sarcomeric proteins (over 800 mutations in 11 different contractile genes) [3]. Many HCM patients, however, lack one of these known mutations. In addition, this is a complex and heterogeneous disease, and the correlations between genotype and phenotype remain poorly understood. To our knowledge, no study has shown that overactivation of FGFR1 is linked to HCM. However, mutations that lead to increased Ras activity, a known mediator of the FGF signaling cascade, are associated with the development of HCM (as well as a host of other cardiac and syndromic defects) [39]. To determine the mechanisms that lead to hypercontractility and the development of HCM in this model, we examined pathways known to be activated by FGFRs (ERK1/2, p38, JNK, Akt, PLCγ1, Stat3, Stat5), but changes tended to be transient and inconsistent, and thus inconclusive. However, expression of the downstream FGF-responsive target genes, *Etv4* and *Etv5*, were consistently and persistently increased. Given the lack of clear activation of one particular pathway, or pathways, it does not appear that these classical downstream signaling pathways are directly involved in the development of hypercontractility or progression of hypertrophy observed in this model. Similar to our findings, there was no increase in phosphorylated ERK or Akt in a Ras-induced HCM mouse model [40], suggesting that a novel or nonclassical pathway was responsible for development of the HCM phenotype. 

We then turned our attention to other possible mechanisms for the development of hypercontractility and HCM, and hypothesized that expression of caFGFR1 resulted in cardiomyocytes that were hypersensitive to sympathetic stimulation. ß-adrenergic receptor activation in cardiomyocytes leads to synthesis of cAMP and activation of protein kinase A (PKA), which phosphorylates downstream targets, such as myofilament proteins (troponin I, myosin binding protein C), ion channels, and calcium handling proteins (phospholamban). Phosphorylation of these targets leads to increased inotropy and lusitropy in cardiomyocytes [33]. However, caFGFR1-expressing hearts showed no increase in PKA activation, and significantly decreased phosphorylation of troponin I (TnI) by one week of DOX induction, suggesting that sympathetic hypersensitization was not the mechanism for the increased contractility. 

Decreased TnI phosphorylation has important implications for the mechanism of phenotype development in this model. TnI is a sarcomeric protein that regulates contraction and relaxation. TnI inhibits the interaction between actin and myosin during diastole, when cytosolic calcium concentration is low. During systole, the calcium concentration rises, calcium binds to troponin C (TnC) and leads to a conformational change in the troponin complex that removes TnI inhibition of cross bridge formation and promotes sarcomere contraction. Phosphorylation of TnI by protein kinase A or C represents an important regulatory mechanism to modulate the contractile properties of the myofilament, as it increases the amount of calcium necessary for activation of contraction (i.e., it decreases the calcium sensitivity of the sarcomere) and increases the rate of calcium dissociation [41,42]. This results in accelerated relaxation and increased crossbridge cycling. Therefore, the decrease in TnI phosphorylation seen in this model would result in increased calcium sensitivity of the sarcomere, leading to contraction at lower calcium concentrations, and ultimately to impaired relaxation. Further evidence that caFGFR1 affects myofilament physiology came from studies of ventricular myocytes isolated from DTG and control hearts. Baseline recordings revealed that DTG myocytes were significantly shorter during diastole than control myocytes, suggesting the presence of a relaxation defect. DTG myocytes also had significantly increased contractility (fractional shortening), suggesting that caFGFR1 leads to intrinsic changes in cardiomyocyte contractile properties. We then subjected these myocytes from DOX-induced DTG and control mice to 2,3-Butanedione monoxime (BDM), an inhibitor of actin-myosin interactions, which significantly increased diastolic sarcomere length in both control and DTG myocytes. While DTG myocytes experienced a greater overall change in sarcomere length, they remained significantly shorter than control myocytes, indicating a partial reversal of the relaxation defect. These data strongly suggest that alterations in cardiomyocyte contractility (specifically relaxation) that result from expression of caFGFR1 are due to intrinsic changes in myofilament physiology. While it is likely that decreased TnI phosphorylation plays a role in the pathophysiology of this model, we did not observe significant changes in p-TnI until seven days of transgene induction, suggesting that other modifications to the contractile apparatus may occur earlier. 

Further studies will be necessary to determine precisely how acute activation of FGF signaling affects myofilament physiology. Mechanisms involved in TnI dephosphorylation are much less clear than those governing phosphorylation, although it has been demonstrated that protein phosphatases 1 or 2A (PP1, PP2A) are capable of dephosphorylating TnI [41,43–46]. Importantly, activation of PP2A has been implicated as a feedback mechanism downstream of FGFR activation [47–49], making this one possible mechanism by which caFGFR1 could have direct effects on myofilament physiology. The potential involvement of protein phosphatases in the pathology of HCM was further implicated in a study utilizing a transgenic mouse model of activated Ras [40]. Regardless of the specific mechanism, decreased TnI phosphorylation and the resulting increased myofilament calcium sensitivity provides a common link between this caFGFR1-mediated HCM and classical models involving sarcomere mutations. At least 62 mutations have been identified in thin filament regulatory proteins (tropomyosin, troponin T, and troponin I), many of which have been shown to increase myofilament calcium binding affinity and one of which prevents PKA-mediated phosphorylation [50,51]. 

Another proposed model for the development and progression of HCM is abnormal intracellular calcium handling [3,4]. Mouse models of HCM have demonstrated decreasing levels of calcium handling proteins, as well as diminished calcium transients and release of calcium from the SR [20,40]. One study even demonstrated that administration of diltiazem before the emergence of hypertrophy was sufficient to maintain normal levels of calcium handling proteins and prevent phenotype development [20]. As a result, we examined the expression levels of various calcium handling proteins, and found that both Serca2 and ryanodine receptor (RyR2) expression were significantly reduced following induction of caFGFR1. Downregulation of Serca2 could potentially result in decreased SR calcium concentration and higher cytosolic calcium concentration. However, published data demonstrate that a reduction in Serca2 expression reduces cardiac contractility and accelerates the progression to heart failure [52], in contrast to the hypercontractile phenotype and lack of systolic dysfunction seen in caFGFR1-induced HCM. Thus, it is more likely that Serca2 is downregulated to compensate for the increased contractility in caFGFR1-expressing hearts. Similarly, decreased RyR2 expression might be expected to decrease calcium-induced calcium release from the SR and thus reduce contractility [53], which suggests that the observed decreased RyR2 expression may be a compensatory change due to the hypercontractility phenotype resulting from caFGFR1 induction. Furthermore, BAPTA-AM, a membrane-permeable calcium chelator, had little effect on sarcomere length in control or DTG myocytes, strongly suggesting that an elevated cytosolic calcium concentration is not responsible for the changes in cardiomyocyte physiology. 

### Clinical applications

To assess the clinical usefulness of this model, we treated DTG mice with drugs shown to be effective in the management of HCM. ß-adrenergic receptor antagonists, such as propranolol, are used for the symptomatic relief of obstructive HCM to decrease the outflow gradient, although the precise mechanisms by which they improve cardiac function and promote reverse remodeling are unknown [36,37]. Additionally, angiotensin receptor antagonists, such as losartan, have been shown to prevent much of the pathological remodeling in animal models of HCM [23], although this drug is contraindicated in patients with LV outflow obstruction due to its effects on blood pressure [38]. Consistent with these data from clinical and animal studies, propranolol significantly reduced both the hypertrophy and dynamic obstruction in DTG mice, while losartan moderately prevented the hypertrophic response, without affecting the obstructive phenotype. These studies demonstrate that this model will be very useful for the evaluation of treatments aimed at preventing, or even reversing, the pathophysiology of HCM.

## Conclusions and Future Directions

The studies presented here have only begun to address mechanisms of adult cardiac FGF signaling, as many significant questions remain. These studies make no assumptions regarding FGF ligands that may be involved in the regulation of cardiomyocyte contractility and hypertrophy, nor do we propose that activation of FGF receptors 2-4 will result in the same phenotype. Additionally, regulation of FGF signaling and activation of intracellular signaling cascades is poorly understood and beyond the scope of this paper. While multiple studies have demonstrated FGF2’s involvement in the development of pathological hypertrophy following injury, limited research has examined the expression or role of other FGF ligands or FGF receptors in the adult heart. 

These studies address consequences of FGF signaling on cardiovascular physiology and remodeling. While many classical mouse models of human HCM exist, our doxycycline-inducible cardiomyocyte-specific caFGFR1 mouse is unique in that it is inducible, reversible, and mediated by a non-sarcomeric protein. This model can be utilized to examine the common pathways that lead to phenotype development, as well as the extent of phenotype prevention and/or reversibility. This model also suggests the possibility that activating mutations in FGFR1 may directly lead to HCM or may modify the phenotype when combined with mutations in other proteins. Finally, perhaps the most striking finding from these studies is that low levels of activated FGFR1 have rapid and potent effects on cardiac contractility without any pathological effects, suggesting that targeted activation of the FGF signaling pathway may be beneficial to patients with heart failure and poor systolic function. Further investigation is necessary to more precisely elucidate the mechanisms responsible for the increased contractility following expression of caFGFR1, as well as pathways involved in the rapid progression from hypercontractility to HCM. 

## Supporting Information

File S1 (DOCX)

Video S1 (MOV)
