# Supplementary material for: Fibroblast Growth Factor Receptor 1 Signaling in Adult Cardiomyocytes Increases Contractility and Results in a Hypertrophic Cardiomyopathy
Source: PLoS One. 2013 Dec 11;8(12):e82979. doi: 10.1371/journal.pone.0082979 (PMC3859602; doi:10.1371/journal.pone.0082979)
Supplement: File S1 — (DOCX) [file pone.0082979.s001.docx]

**Supplemental Material**

**Supplemental Tables**

**Supplemental Table S1. Hemodynamic analysis of double transgenic and littermate control mice following 24 hours on DOX chow.**

| **Parameter*** | **αMHC-rtTA** | **αMHC-rtTA, TRE-caFGFR1** | **T-test** |
| --- | --- | --- | --- |
| Number of mice | 4 | 4 | ***P value*** |
| Heart rate (bpm) | 366 ± 104 | 332 ± 69 | NS |
| MAP | 64.9 ± 3.1 | 61.6 ± 1.6 | NS |
| ESP (mmHg) | 85.7 ± 7.0 | 79.4 ± 4.1 | NS |
| EDP (mmHg) | 5.8 ± 2.4 | 6.4 ± 1.9 | NS |
| ESV (µL) | 31.7 ± 9.0 | 18.2 ± 6.5 | 0.05 |
| EDV (µL) | 51.2 ± 9.0 | 31.8 ± 5.7 | 0.01 |
| EF (%) | 38.8 ± 8.2 | 43.8 ± 16.2 | NS |
| dP/dt-max (mmHg/s) | 4701 ± 739 | 4436 ± 1593 | NS |
| dP/dt-min (mmHg/s) | -4307 ± 791 | -3503 ± 952 | NS |
| *tau* Glantz | 16.7 ± 2.8 | 19.5 ± 6.3 | NS |

*Note absence of differences in all parameters except end systolic and end diastolic volumes (ESV, EDV). bpm, beats per minute; MAP, mean arterial pressure; ESP, end systolic pressure; EDP, end diastolic pressure; EF, ejection fraction; dP/dt, change in pressure over change in time.

**Supplemental Table S2.** **Primary and secondary antibodies utilized for Western blotting.**

| **Antibody** | **Catalog #** | **Dilution** | **Secondary** | **Catalog #** | **Dilution** |
| --- | --- | --- | --- | --- | --- |
| Mouse anti-Serca2 | Thermo Scientific #2A7-A1 | 1:2000 | Goat anti-mouse IgG HRP | Santa Cruz sc-2055 | 1:10,000 |
| Mouse anti-human c-myc | DSHB 9E10 | 1:2000 | Goat anti-mouse IgG HRP | Santa Cruz sc-2055 | 1:5000 |
| Rabbit anti-mouse phospho-ERK1/2^T202/Y204^ | Cell Signaling #4376S | 1:1000 | Goat anti-rabbit IgG HRP | Santa Cruz sc-2301 | 1:10,000 |
| Rabbit anti-mouse ERK1/2 | Cell Signaling #9102S | 1:1000 | Goat anti-rabbit IgG HRP | Santa Cruz sc-2301 | 1:10,000 |
| Rabbit anti-mouse phospho-Akt^S473^ | Cell Signaling #4058S | 1:1000 | Goat anti-rabbit IgG HRP | Santa Cruz sc-2301 | 1:10,000 |
| Rabbit anti-mouse Akt | Cell Signaling #9272S | 1:1000 | Goat anti-rabbit IgG HRP | Santa Cruz sc-2301 | 1:10,000 |
| Rabbit anti-PLCg1 [pY^783^] | Invitrogen #44-696G | 1:500 | Goat anti-rabbit IgG HRP | Santa Cruz sc-2301 | 1:2000 |
| Rabbit anti-PLCg1 (D9H10) XP | Cell Signaling #5690 | 1:500 | Goat anti-rabbit IgG HRP | Santa Cruz sc-2301 | 1:5000 |
| Rabbit anti-FGF receptor 1 XP | Cell Signaling #9740 | 1:1000 | Goat anti-rabbit IgG HRP | Santa Cruz sc-2301 | 1:2000 |
| Rabbit anti-phospho-Stat3 (Tyr705) XP | Cell Signaling #9145 | 1:2000 | Goat anti-rabbit IgG HRP | Santa Cruz sc-2301 | 1:2000 |
| Mouse anti-Stat3 (124H6) | Cell Signaling #9139 | 1:2000 | Goat anti-mouse IgG HRP | Santa Cruz sc-2055 | 1:5000 |
| Rabbit anti-Stat5 (Tyr694) (C71E5) | Cell Signaling #9314 | 1:2000 | Goat anti-rabbit IgG HRP | Santa Cruz sc-2301 | 1:2000 |
| Rabbit anti-phospho Troponin I (Ser23/24) | Cell Signaling #4004 | 1:2000 | Goat anti-rabbit IgG HRP | Santa Cruz sc-2301 | 1:10,000 |
| Mouse anti-Troponin I | DSHB TI-1 | 1:2000 | Goat anti-mouse IgG HRP | Santa Cruz sc-2055 | 1:10000 |
| Rabbit anti-phospho-p38 (Thr180/Tyr182) (D3F9) Xp | Cell Signaling #4511 | 1:2000 | Goat anti-rabbit IgG HRP | Santa Cruz sc-2301 | 1:5000 |
| Rabbit anti-p38 | Cell Signaling #9212 | 1:2000 | Goat anti-rabbit IgG HRP | Santa Cruz sc-2301 | 1:5000 |
| Mouse anti-phospho-SAPK/JNK (Thr183/Tyr185) | Cell Signaling #9255 | 1:2000 | Goat anti-mouse IgG HRP | Santa Cruz sc-2055 | 1:10,000 |
| Goat anti-JNK1/2 | Santa Cruz sc-474 | 1:1500 | Donkey anti-goat IgG HRP | Santa Cruz sc-2304 | 1:2000 |
| Rabbit anti-phospho-PKA | Cell Signaling #5661 | 1:1000 | Goat anti-rabbit IgG HRP | Santa Cruz sc-2301 | 1:10,000 |
| Rabbit anti-PKA | Cell Signaling #5842 | 1:1000 | Goat anti-rabbit IgG HRP | Santa Cruz sc-2301 | 1:10,000 |
| Rabbit anti-phospho-phospholamban | Cell Signaling #8496 | 1:2000 | Goat anti-rabbit IgG HRP | Santa Cruz sc-2301 | 1:20,000 |
| Rabbit anti-phospholamban | Cell Signaling #8495 | 1:2000 | Goat anti-rabbit IgG HRP | Santa Cruz sc-2301 | 1:20,000 |
| Rabbit anti-b-tubulin | Abcam #ab6046 | 1:50,000 | Goat anti-rabbit IgG HRP | Santa Cruz sc-2301 | 1:50,000 |

**Supplemental Table S3. Taqman® gene expression assays utilized for qRT-PCR.**

| **Gene** | **Applied Biosystems (ABI) Taqman® Gene Expression Assay** |
| --- | --- |
| *Fgfr1 (tyrosine kinase domain)* | Mm0125485_g1 |
| *Anp (Nppa)* | Mm01255748_g1 |
| *Bnp (Nppb)* | Mm00435304_g1 |
| *bMHC (Myh7)* | Mm00600555_m1 |
| *Col1a1* | Mm00801666_g1 |
| *Col3a1* | Mm01254476_m1 |
| *Etv4 (pea3)* | Mm00476696_m1 |
| *Etv5 (erm)* | Mm00465816_m1 |
| *NCX1(Slc8a1)* | Mm01232254_m1 |
| *Ryr2* | Mm00465877_m1 |
| *Serca2 (Atp2a2)* | Mm01201431_m1 |
| *Hprt* | Mm01545399_m1 |

**Supplemental Figures**


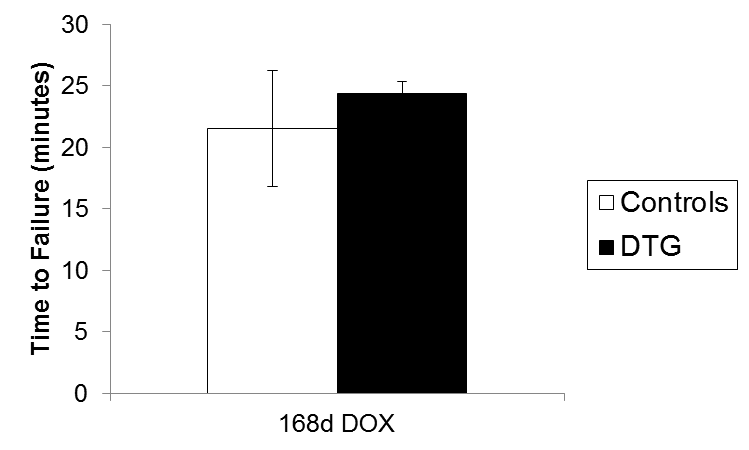


**Supplemental Figure S1. Chronic induction of caFGFR1 and significant concentric hypertrophy does not cause exercise intolerance in double transgenic mice.** Twelve- to 14-week-old mice were fed DOX chow to induce caFGFR1 expression for 168 days (six months), and then subjected to an acute treadmill stress test to determine if the presence of severe hypertrophy resulted in exercise intolerance. Double transgenic mice (DTG) were capable of running for the same amount of time as their single transgenic littermate controls (n_DTG_=3, n_control_=4). Error bars = standard deviation.


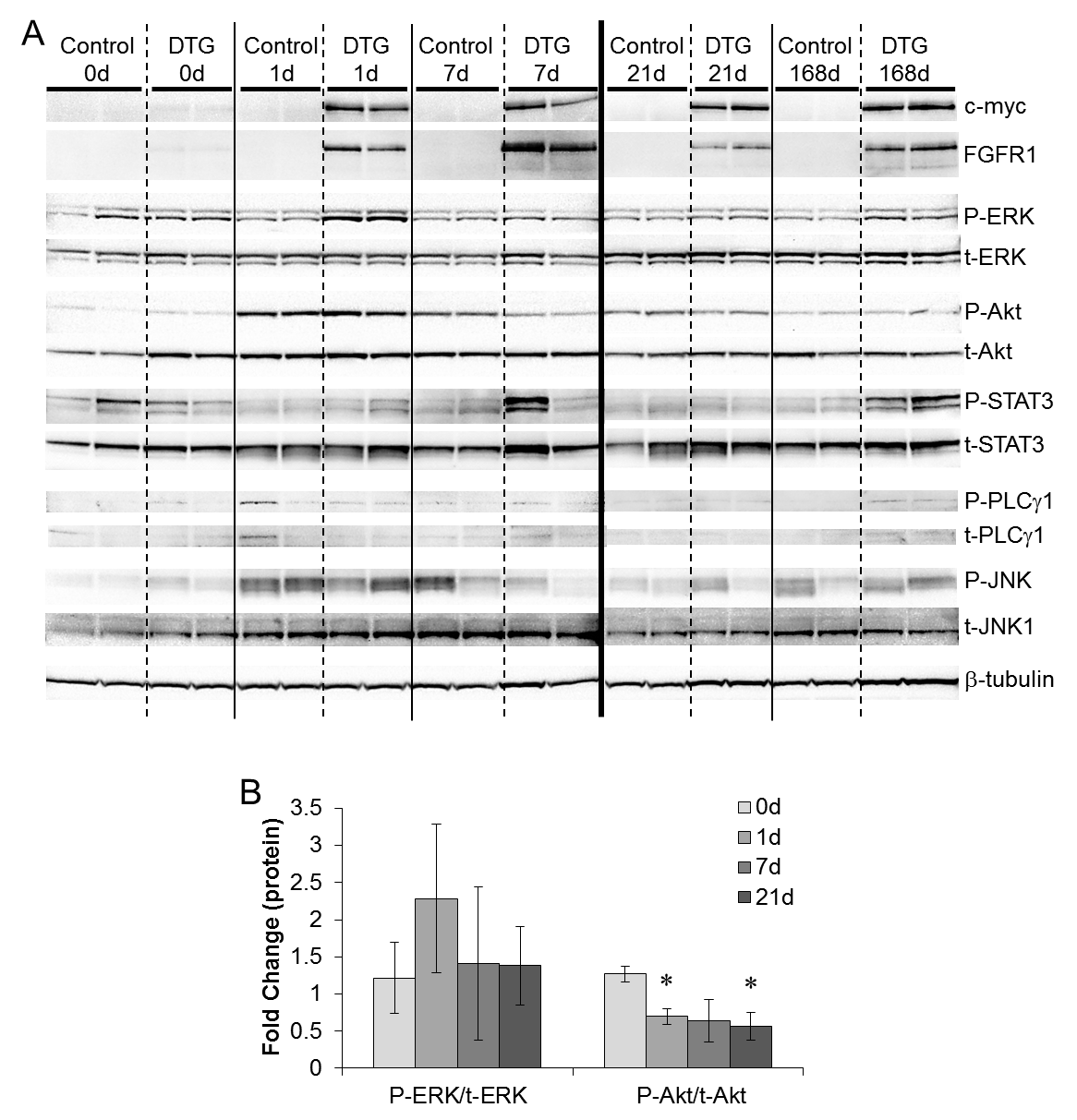


**Supplemental Figure S2.** **Induction of caFGFR1 results in transient ERK1/2 activation and sustained downregulation of Akt activation.** (A) Representative Western blots examining signaling pathways downstream of the FGF receptor. Note that most pathways examined, as well as p38 and Stat5 (not shown), showed considerable variability. (B) Densitometric analysis of multiple independent Western blots demonstrated a transient and insignificant 2-fold increase in ERK1/2 activation, and a sustained decrease in Akt activation. DTG (shown): n_0d_=5, n_1d_=3, n_7d_=8, n_21d_=6; controls (not shown): n_0d_=2, n_1d_=2, n_7d_=4, n_21d_=3. Error bars = standard deviation. *p<0.05.


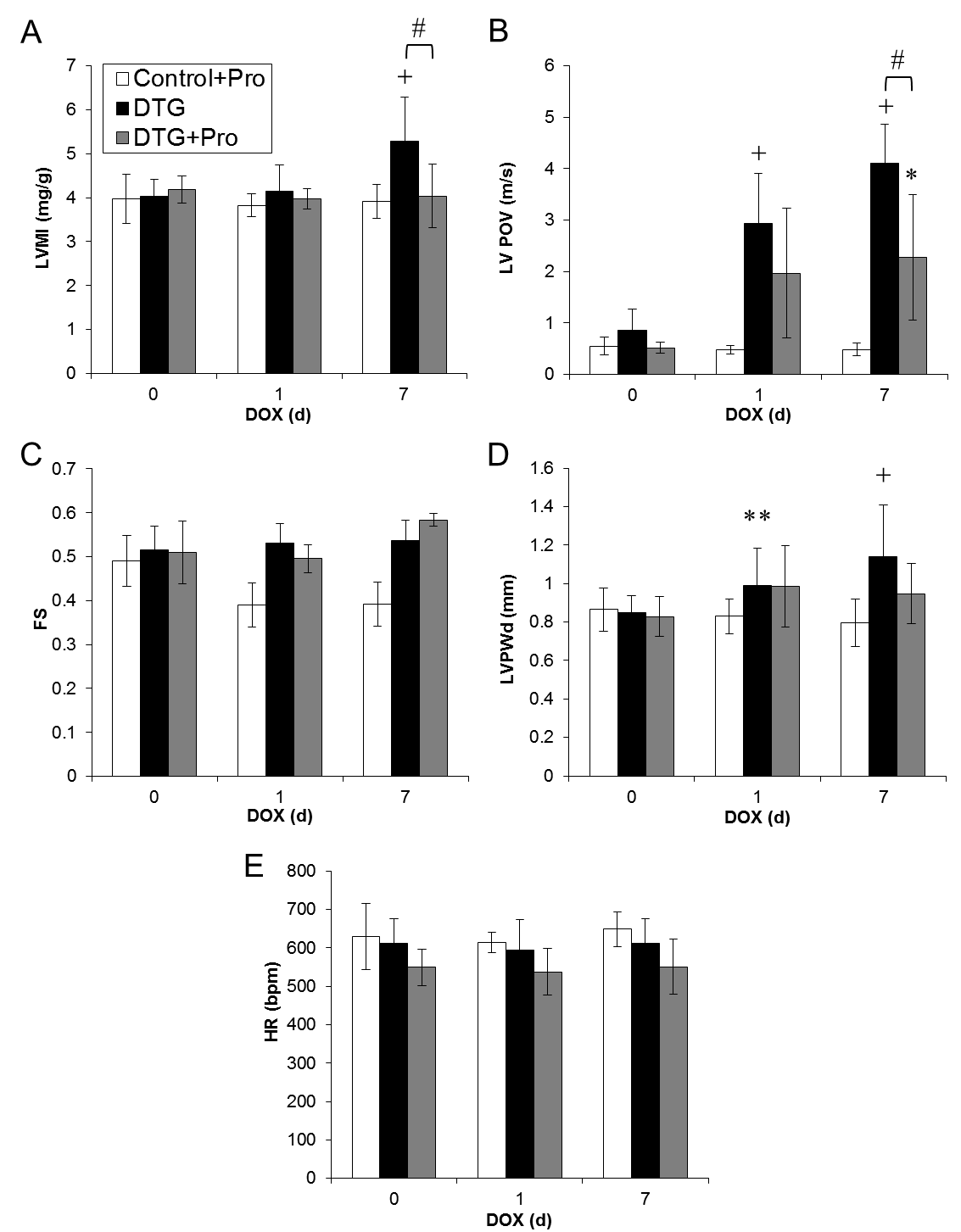


**Supplemental Figure S3. Propranolol (Pro) blocks development of hypertrophy and decreases outflow velocity in DTG mice.** (A) LV mass index (LVMI) in propranolol-treated DTG (DTG + pro) mice was comparable to controls (Control + Pro) and significantly lower than untreated DTG mice induced for one week. (B) LV peak outflow velocity (LV POV) was also significantly reduced in propranolol-treated DTG mice compared to DTG controls induced for one week, although it was still significantly elevated compared to baseline (0d DTG + Pro). (C) No significant changes were observed in fractional shortening (FS), although there was a trend towards enhanced systolic function in propranolol-treated DTG mice. (D) Propranolol treatment led to a moderate reduction in LV diastolic posterior wall thickness (LVPWd) following one week of caFGFR1 expression. (E) No significant changes in heart rate were observed with propranolol treatment. Error bars = standard deviation. n_DTG+Pro_=5, n_control+Pro_=3, n_DTG,0d_=51, n_DTG,1d_=9, n_DTG,7d_=14. *p<0.05, **p<0.01, +p<0.001 vs. corresponding baseline (0d) utilizing paired t-test. #p<0.05 DTG+pro vs. untreated DTG utilizing unpaired t-test.


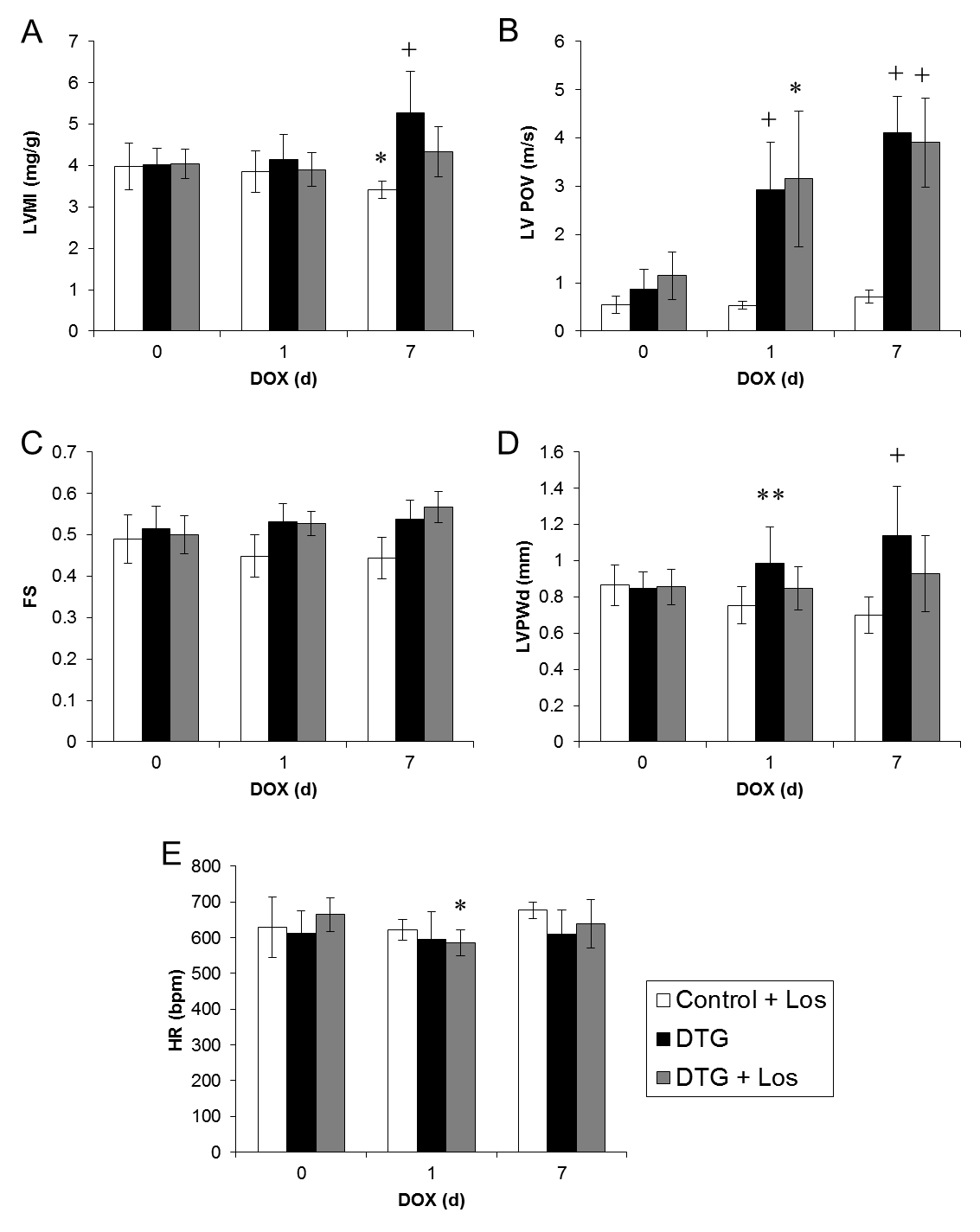


**Supplemental Figure S4. Losartan (Los) lessens hypertrophy development but does not affect outflow velocity in DTG mice.** (A) Losartan-treated DTG (DTG + Los) mice did not experience a significant increase in LVMI following one week on DOX. (B) Losartan-treated DTG mice induced for one day and one week had significantly increased LV peak outflow velocities comparable to untreated DTG mice. (C) There were no significant differences in systolic function in losartan-treated DTG mice. (D) Losartan treatment led to a moderate reduction in LVPWd following one week of caFGFR1 expression. (E) No significant changes in heart rate were observed with losartan treatment. Error bars = standard deviation. n_DTG+Los_=6, n_control+Los_=3, n_DTG,0d_=51, n_DTG,1d_=9, n_DTG,7d_=14. *p<0.05, **p<0.01, +p<0.001 vs. corresponding baseline (0d) utilizing paired t-test. #p<0.05 DTG+pro vs. untreated DTG utilizing unpaired t-test.

**Supplemental Video Legend**

**Supplemental Video 1. Induction of caFGFR1 enhances cardiac contractility and leads to concentric hypertrophy**. Note the enhanced cardiac function following 24 hours of DOX (1d DTG), before the emergence of hypertrophy. Also note the progressive enlargement of the LV and the absence of systolic dysfunction throughout six months of induction (168d DTG).
